# Prevalence of recurrent aphthous ulceration experience in patients attending Piramird dental speciality in Sulaimani City

**DOI:** 10.4317/jced.51042

**Published:** 2013-04-01

**Authors:** Mustafa J. Abdullah

**Affiliations:** 1 B.D.S., M.Sc.Oral Medicine, Assistant lecturer in the Oral Medicine Clinic of the school of dentistry, University of Sulaimani, Kurdistan region, Iraq

## Abstract

Objective: The aim of this study is to report the prevalence and risk factors of recurrent aphthous ulceration (RAU) in patients attending Piramird dental speciality for seeking dental treatment. Study design: A cross-sectional survey was carried out among patients (n=1100) who were visiting the department of oral medicine at Piramird dental speciality center in Sulaimani from December 2011-February 2012. The age range of the patients were between 10-79 years, with mean age of (34.27±14.14). 446 (44.6%) of participants were males and 554 (55.4%) were females, with male/female ratios of 0.80:1. All individuals had to answer specific questions including personal data (age, sex), level of education, occupation and smoking habit; etc. Additional questions were related to the risk factors that might be related to the condition. Chi Square test was used to analyze the data. Result: The life time prevalence of RAU experience was 28.2% (n=282). It was highly significantly more common among females (31.76%) (p<0.004). The most commonly affected age group was 20-29 years (36.28%). The highest prevalence of RAU experience was seen among mere students (36.8%); Among non smokers there were highly significantly more patients with RAU experience (30%) than in heavy smoker patients (12.22%), (p=0.000). 34.4% of patients had family history of RAU. Lips and buccal mucosae were the commonest sites of ulcerations (73.10%), and the major risk factor was stress (43.3%). Conclusion: This study has provided information about the epidemiologic aspects of recurrent aphthous ulceration, Based on the finding of this study, RAU is a common, recurrent painful oral ulceration. This study point to the importance of a thorough history taking to identify the patient’s main risk factors to get preventive measures, therefore treatment will be tailored for each patient accordingly. And the author concluded that stress was the major risk factor, thus, stress-management interventions suggested to be beneficial in reducing RAU recurrence episodes.

** Key words:**Recurrent aphthous ulceration, prevalence, stress.

## Introduction

Recurrent aphthous ulceration (RAU) is the most common inflammatory ulcerative condition of the oral mucosa (1) with a high prevalence among women (2).

RAU occurs in the non-keratinized areas as lips, tongue, buccal mucosa and soft palate (3). They are usually painful, shallow round ulcers with an erythematous halo covered by a yellowish-gray fibromembranous layer (4).

Many suggestions have been proposed but the etiology of recurrent aphthous ulceration is still controversial (5) and its occurrence is related to a range of factors (1); precipitated factors include stress, physical or chemical trauma, food sensitivity, and genetic predisposition (6). The still unclear etiology has resulted in treatments that are largely empiric and aimed at symptom reduction. The aim of this study is to report the prevalence and risk factors of RAU in patients attending Piramird dental speciality for seeking dental treatment.

## Patient and Methods

A cross-sectional survey was carried out among patients (n=1100) who were visiting the department of oral medicine at Piramird dental speciality center in Sulaimani along 3 successive months (December 2011-February 2012) for seeking dental treatment. This research was approved by the Committee of Ethics in Research of the University of Sulaimani and Piramird health center. According to Declaration of Helsinki, signed consent forms were obtained from all participants before conducting the study (7).

All individuals underwent an interview in which they had to answer specific questions include personal data (age, sex), Level of education [(level 0 (illiterant), level 1 (primary school), level 2 (secondary school), level 3 (preparatory school), level 4 (bachelors and institution), level 5 (diploma, master, and PhD)], Occupation (student, house wife, blue collar worker, white collar worker, retired people, general worker, unemployed people and others). and smoking habit, subjects were classified into 3 categories according to their reported smoking habit, namely, “smoker” (smokes every day), “former smoker”, and “never been smoker”. “Former smoker” and “never been smoker” were defined as “non-smokers” in data analyses. Furthermore, smoking status of current smokers was determined based on the average number of cigarettes smoked throughout life (average of daily smoked cigarettes multiplied by duration of habits in days). Cigarette smokers were classified into light smokers (smoking 1-10 cigarettes/day), moderate smokers (smoking 11-20 cigarettes/day), and heavy smokers (smoking 20 cigarettes/day) (8).

The participants were asked whether they had oral ulcers or oral aphthosis in his or her life. After describing the aphthosis to them as a round recurrent painful ulcer with a white/ yellow center. Additional questions were including ulcer history, shape, location, duration, family history, number (single or multiple), painful or not. Finally, risk factors that might be related to the condition were reported, including stress, hormonal factors, relation to certain type of food, history of systemic diseases and medications.

Statistical analysis was performed using SPSS program version 16. Associations between categorical variables were tested using chi-square test; Statistical significance was set at P < 0.05.

## Result

The number of subjects invited to participate were 1100 subjects. But 100 of them reject to participate; therefore, the final number was 1000 participant. The age range of the patients was between 10-79 years, with mean age of (34.27±14.14). 446 (44.6%) of participants were males and 554 (55.4%) were females, with male/female ratios of 0.80:1. Patients were divided in to 7 age groups (10 years for each interval). Accordingly more than half of patients 552 (55.2%) were in the 2nd and 3rd age group ( Table 1).

**Table 1:**
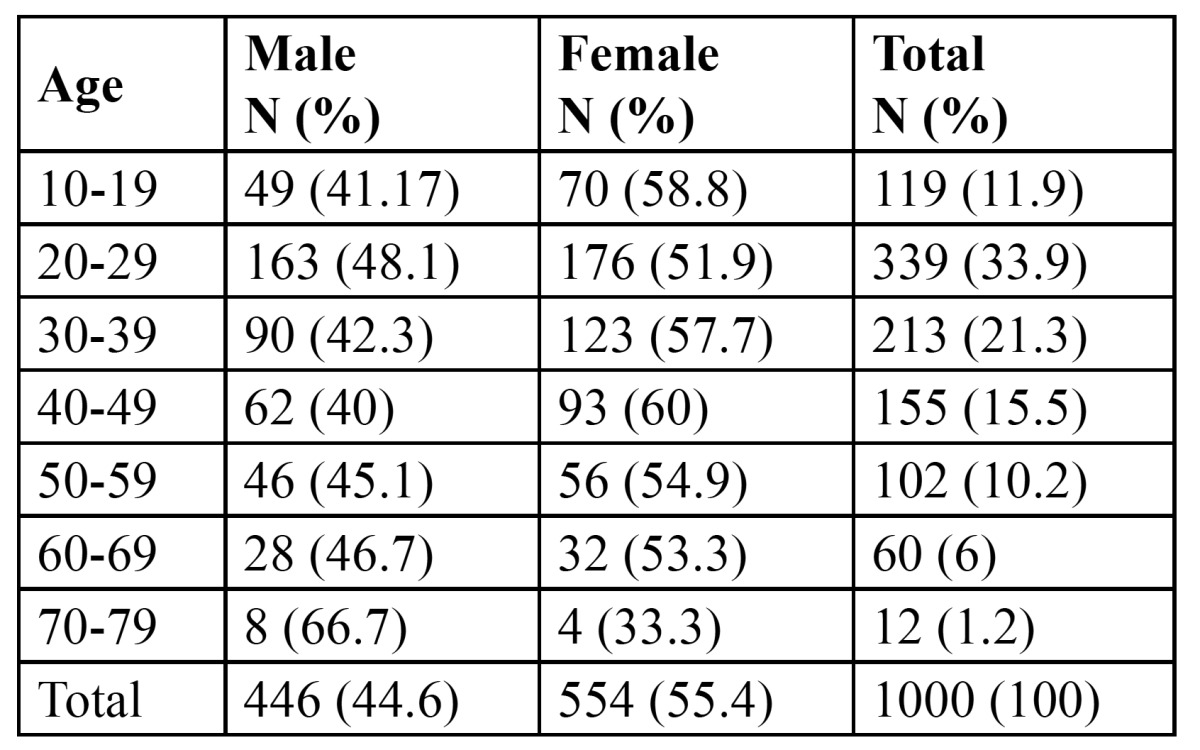
Sample distribution by age and sex.

The prevalence of RAU experience was 28.2% (n=282). It was highly significantly more common among fe-males 31.76% (p<0.004). The most commonly affected age group was 20-29 years (36.28%), followed by 30-39 year old. These two age groups showed the highest prevalence of RAU experience for both male and females. The smallest prevalence was observed in the oldest age group 70-79 years (8.33%) (p<0.04) ( Table 2).

**Table 2:**
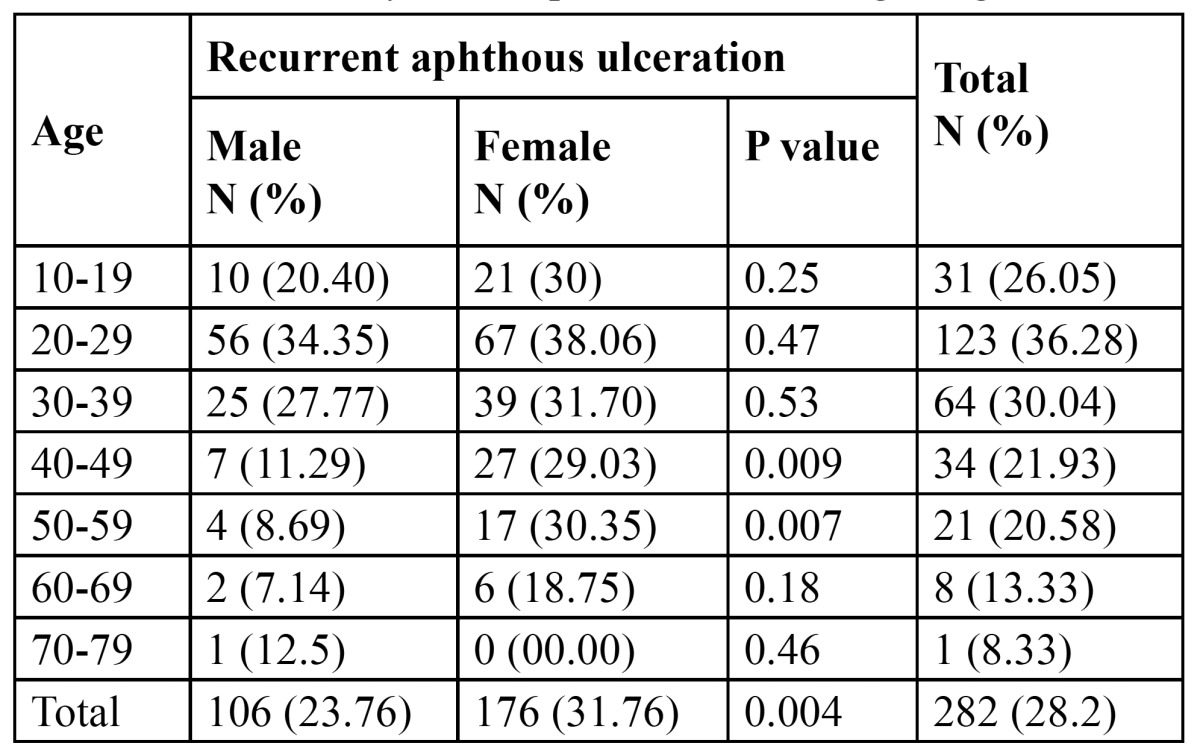
Prevalence of RAU experience according to age and sex.

Prevalence of RAU was increased as the level of education is increased (level 0=20.75%, level 4= 35.22%, P<0.009, level 5=75%, p<0.01). Regarding occupation of patients; the highest prevalence of RAU experience was seen among mere students (36.8%); followed by house wife (30.49%), and blue collar workers (25%), among non smokers there were highly significantly more patients with RAU experience (30%) than in heavy smoker patients (12.22%), (p=0.000) ( Table 3).

**Table 3:**
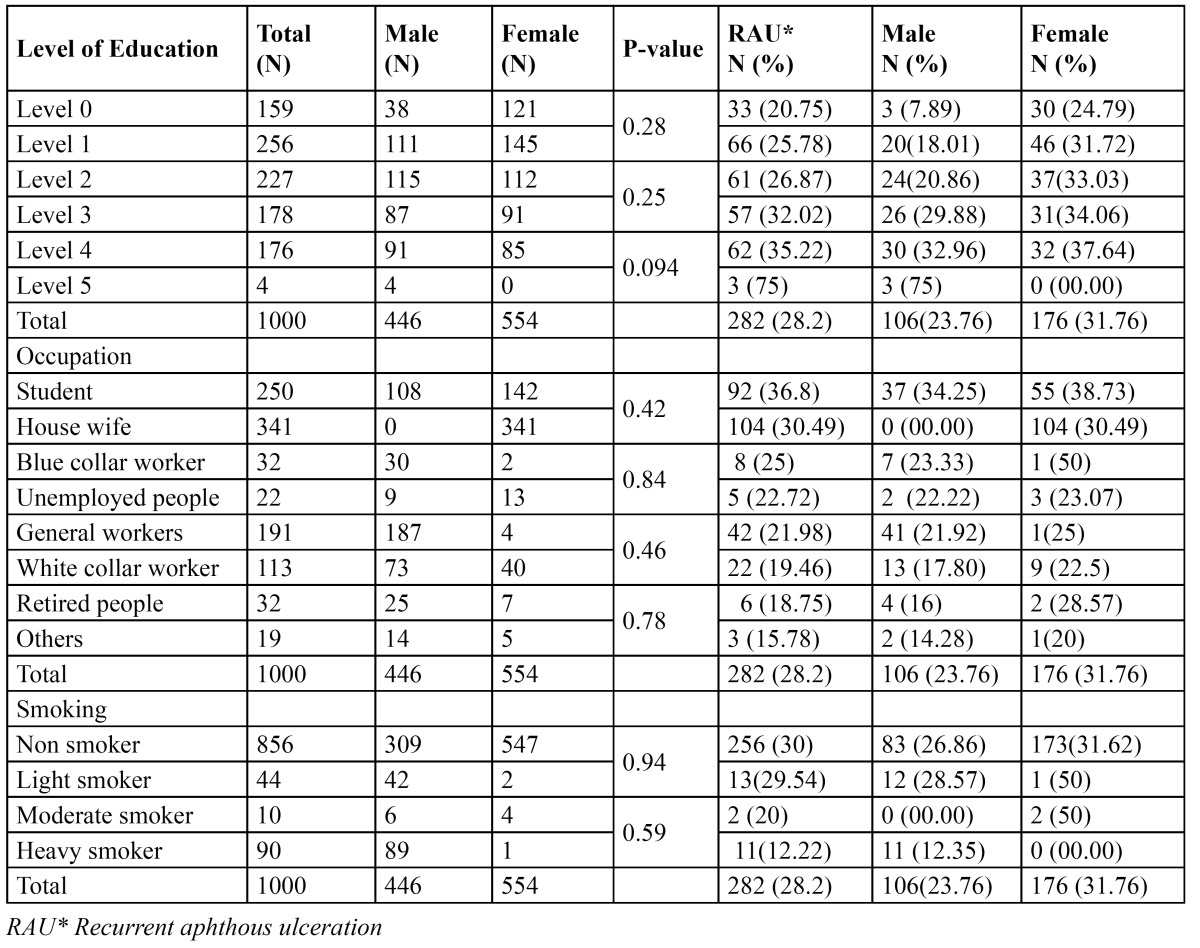
Prevalence of recurrent aphthous ulceration experience according to level of education, occupation and habit of smoking.

Regarding information related to RAU, approximately 34.4% of patients reported that other family members were suffered previously from RAU. Lips and buccal mucosae were the commonest sites of ulcerations (73.10%), followed by tongue (18.87), floor of mouth (4.12%) and gingiva (3.90). 62.41% of patients reported that their RAU were circular in shape. More than two third of patients (76.2%) reported that ulcerations were single ulcerations while about 23.8% of patients reported as multiple ulcerations. About 75.5% of patients reported that the ulcers were painful and 79.1% claimed that the ulcerations interfered with food eating and swallowing. The duration of ulceration was reported to be less than a week for about 56.7% of patients while about 40.8% of patients reported that the ulceration extended up to two weeks and only minority (2.5%) reported that the duration of ulceration extended longer than two weeks.

Approximately 43.3% of patients reported that their RAU were related to stress; and 29.1% were related to trauma, 7.1% related their ulceration to eating certain type of food, 5.31% experience ulceration during episodes of common cold, 2.48% reported that their ulceration were related to menstruation. Regarding systemic diseases, the majority (21.98%) of patients had gastrointestinal diseases (disease of colon (14.89%), and Peptic ulceration (7.09%)), hypertension constitute 7.80% of all patients with reported history of RAU. Concerning type of medications that the patients used, Majority of patients with gastrointestinal disturbances were used symptomatic drug treatment for short period of time, hypertensive patients (3.54%) using (tenormin®, losartan potassium, prazosin, thiazide, frusemide, amlodipine, inalapril) and 4.60% of patients with reported history of RAU were used pain killers (panadol®, naproxen, aspergic, voltaren®) ( Table 4).

**Table 4:**
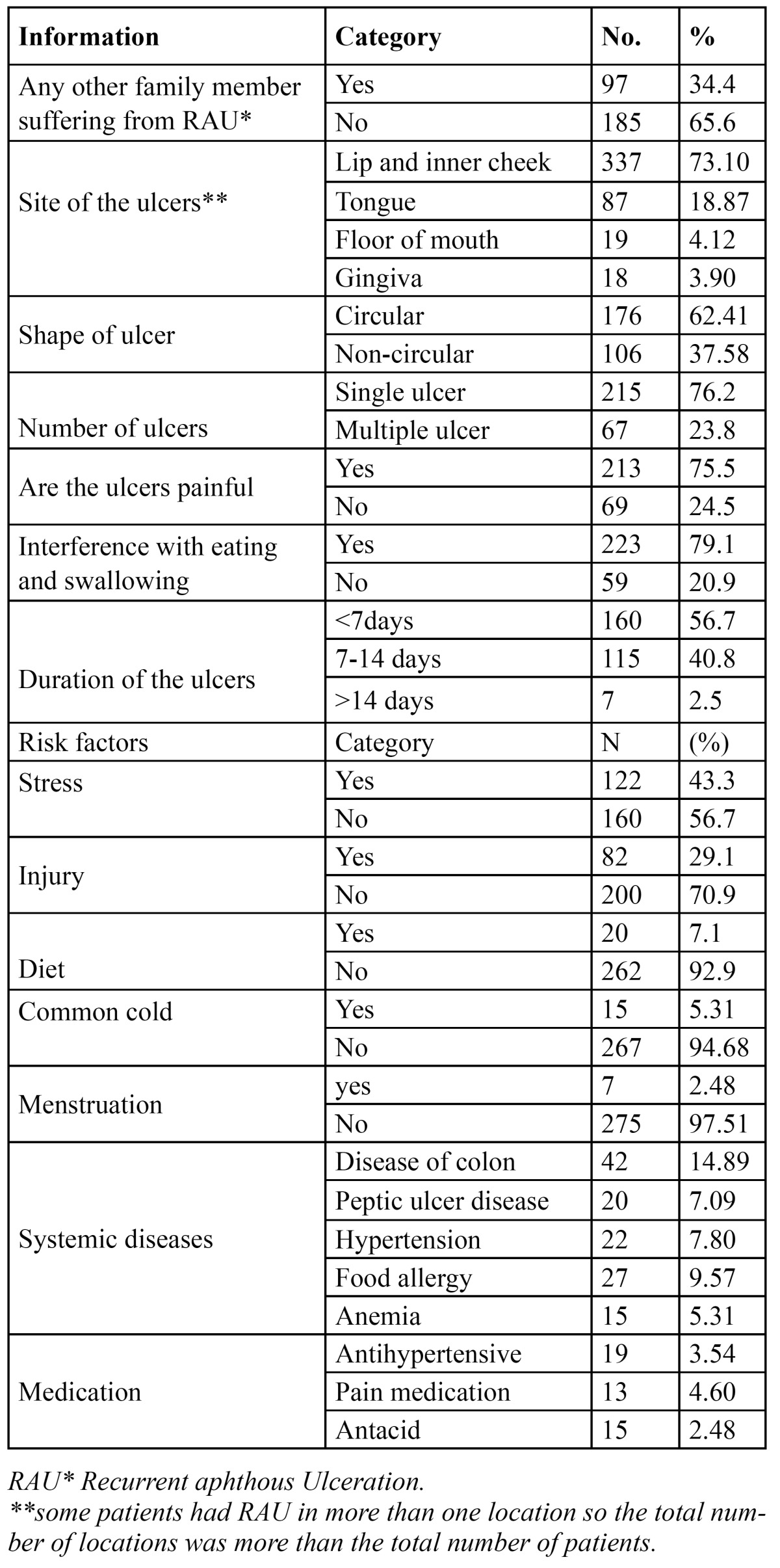
Distribution of informations and the risk factors for the 282 patients that reported history of recurrent aphthous ulceration.

## Discussion

Epidemiological studies performed over the past few years have shown considerable variation in the prevalence of RAU among different regions throughout the world. The prevalence range among differing populations has been documented as 5-66% (4,9,10).

In this study the life time prevalence of RAU was (28.2%). When compared with other studies, the figure was around the same value. It was reported (25.2%) in Iran (11), (25.5%) in Turkey (12) and 17.7% in Sweden (13) but lower than the prevalence reported in Jordan (78.1%) (2). These variations could be explained due to the fact that in this type of study you depend on patients memory about RAU in his or her life as patients were asked “ have you ever suffered from such a lesion in your life” so this type of study depends mainly on patients recollection of the condition in the past, or probably the patient mix RAU with other type of ulcerations, different methodologies used (whether it is registered when the lesion is present, or it s is done through the clinical history (14), socioeconomic level (15), genetic predisposition, life style of patients and stress (2).

RAU history of experience was highly significantly more common among females than males (p<0.004). Several other studies showed higher prevalence of RAU among females (2). In relation to the female predisposition to RAU, some authors have suggested that this association is related to hormonal rates (16).

As a minority of women with RAU have cyclical oral ulceration related to the luteal phase of the menstrual cycle (17,18) and also a decrease in its incidence during pregnancy, thus relating the episodes of RAU to progesterone levels (19). On the contrary, Rivera-Hidalgo et al. (4) found a higher prevalence of RAU among males, although without statistical significance.

There is some evidence that the disease has a higher prevalence in younger adults, decreasing in both incidence and severity with age (20). In this study, the most commonly affected age group was 20-29 years, the prevalence decreased as the age is increased. This result is in accordance with the finding of Davatchi et al. (11) in Iran.

Educational level and occupational status had great impact on the prevalence of RAU; prevalence of RAU is increased as the level of education is increased and the highest prevalence among mere students. This finding supports the role of stress and anxiety in occurrence of RAU among educated patients, especially during school exam.

An inverse relationship between tobacco use and the appearance of RAU has been observed in the literature (21). Some researchers thought that smoking has protective effect and this protective effect is related to the increased keratinization of the oral mucosa in smokers and that this keratin layer acts as a mechanical and chemical barrier against trauma or microbes (22). Few investigators suggested that smokers may be less psychologically stressed than nonsmokers and that some psychological trigger might affect RAU development (6). The association found in this study between heavy cigarette smoking and less prevalence of RAU suggests that smoking may play a role in preventing the occurrence of RAU. Although such lower prevalence of RAU in the heavy smokers shouldn’t encourage smokers who suffer from RAU to increase their consumption.

Regarding family history of RAU, a genetic predisposition for the development of aphthous ulcer is strongly suggested, as in one study about 40% of patients have a family history and these individuals develop ulcers earlier and are of more severe nature (1). Various associations with HLA antigens and RAU have been reported. These associations vary with specific racial and ethnic origins (23). A number of several other studies have shown a familiar trend in the development of RAU (24) and the correlation is also greater in identical twins (21), demonstrating the existence of a genetic influence in the episodes. Similarly, in this study, 34.4% of patients reported that other family member suffered previously from RAU.

This study demarcates that lips and buccal mucosa were the commonest sites of RAU which are in accordance with the finding of Safadi (2) in Jordan. This may probably because these 2 sites are more prone to trauma.

Psychological stress as a triggering factor for RAS has already been mentioned in the literature, and is typically observed during stressful situations (25). In a study done by Gallo et al. (26), 68% of patients reported that the occurrence of RAU was associated with some of the aforementioned situations, particularly changes in life such as family problems, new job, and new position at the job or new location of residence. Some investigators have speculated that anxiety could lead to parafunctional oral habits, including lip and cheek biting, and that those physical traumas may initiate the ulcerative process in susceptible individuals (26). In this study 43.3% of patients reported that their RAU were related to stress, and this is in agreement with other studies where stress has been emphasized as a causative factor in RAU.

 In this study trauma was the second most common risk factor for RAU, yet a review of the literature shown that; Injury was the second factor mentioned as being responsible for the development of RAU (3,16), injuries caused by anesthetic injection, sharp foods, brushing and/or dental treatment may trigger the RAU. However, according to Barrons (24), edentulous patients are not susceptible to RAU due to prosthetic injuries, probably because of the greater keratinization of the alveolar ridge that restricts the wound to the underlying tissue.

In this study, 7.1% of patients related the condition to eating certain types of food. Thereby reinforcing the concern raised by other authors, who have found that the presence of allergies or sensibility to foods or tissue irritation from certain substances in these foods are closely related to the occurrence of RAU (27).

Nowadays RAU may also related to common cold, the most widely accepted theory explain this relation is related to immunological changes, which reacts nonspecifically to unknown antigens, but this response also could be related to the presence of microorganisms (28). Various microorganisms have been tested, but none have been unequivocally incriminated in RAU. In this study, about 5.31% of patients related their ulceration to common cold.

Furthermore, small percentage of females reported that their RAU were related to menstruation. Conflicting reports exist regarding such association. Studies state association of oral ulceration with onset of menstruation or in the luteal phase of the menstrual cycle (23). Mc Cartan et al. (29) in 1992 established no association between RAU and premenstrual period, pregnancy, or menopause.

Regarding systemic diseases, 21.98% of patients were reporting history of RAU with gastrointestinal diseases. Previous literature, remarked the association of the lesion in some individuals with systemic conditions such as gastrointestinal disease (30).

Certain drugs have been associated with development of RAU; these include angiotensin converting enzyme inhibitor captopril, gold salts, nicorandil, phenindione, phenobarbital, and sodium hypochloride. NSAIDS such as propionic acid, diclofenac, and piroxicam may also cause oral ulceration similar to RAU (20). In this study; small percentages of patients used pain medications, and antihypertensive medications.
